# Stop Thinking! I Can't! Do Attentional Mechanisms Underlie Primary Dysfunctional Breathing?

**DOI:** 10.3389/fphys.2018.00782

**Published:** 2018-06-22

**Authors:** Laís S. Vidotto, Marcelo Bigliassi, Mandy O. Jones, Alex Harvey, Celso R. F. Carvalho

**Affiliations:** ^1^Department of Clinical Sciences, Brunel University London, Uxbridge, United Kingdom; ^2^Department of Life Sciences, Brunel University London, Uxbridge, United Kingdom; ^3^Department of Physical Therapy, University of São Paulo, São Paulo, Brazil

**Keywords:** attention, respiratory medicine, psychosomatic medicine, respiration disorders, attentional control

## Definition of primary dysfunctional breathing

A mystery surrounds the realm of pulmonary medicine; a respiratory condition named by researchers as *primary dysfunctional breathing* (PDB; Jones et al., 2013). This condition is frequently manifest in the worldwide population but still requires further investigation (Boulding et al., 2016). PDB has been characterized as a breathing disorder that ultimately results in erratic patterns of respiration with no evident anatomical abnormalities or physiological alterations, but that appears to be linked to one's emotional state and situational triggers, e.g., anxiety, suppressed, anger and grief (Gilbert, 1998). The symptoms presented by patients with PDB commonly vary from “air hunger” (i.e., breathlessness) to dizziness and palpitations (Jones et al., 2013). These physical sensations can sometimes initiate for inexplicable reasons and may lead to a consequent deterioration of health-related quality of life (Thomas et al., 2003). Given the extreme difficulty to understand and identify PDB, clinicians all over the world have repeatedly provided patients with uncertain diagnosis (cf. Hagman et al., 2008). Unfortunately, the methods used by physicians and health professionals to detect this condition can vary considerably, be imprecise and frequently involve the use of inappropriate assessment tools (e.g., the use of Nijmegen Questionnaire only; van Dixhoorn and Folgering, 2015), and/or subjective measures of the respiratory pattern (e.g., visual inspection; Chapman et al., 2016). The under-and misdiagnosis of PDB can be attributed to the lack of high quality studies into the condition and to the absence of validated diagnostic tools to be used both in research and clinical practice.

PDB is recurrently associated with psychological factors such as stress and anxiety. In actuality, researchers have recently proposed that this could be the root cause of PDB (i.e., acting as a mediator; Courtney, 2016). This opinion article proposes a paradigm shift to further the understanding of PDB. It presents the hypothesis that some of the mechanisms that underlie the physical sensations associated with this condition can only be explained from a neuroscientific standpoint. In order to explore the sequence of psychophysiological mechanisms behind this approach, we firstly need to understand the perceptual responses associated with PDB. Accordingly, compelling evidence indicates that some of the commonly reported symptoms associated with this respiratory disorder are primarily triggered by emotional responses (e.g., psychological stress; Han et al., 1996; Courtney et al., 2011). In such instances, the problem we have to solve as scientists is to unravel the cerebral mechanisms that underpin the conversion of emotional reactions into functional and physiological alterations. For example, how could emotionally charged situations lead to breathlessness? Why are the cardiovascular and respiratory systems so easily affected by psychological responses? These are some of the questions that remain unanswered and that can only be explored if researchers expand their investigation beyond peripheral physiological indices such as resting carbon dioxide. It is important to emphasize that the theoretical propositions of this paper are not applicable to secondary dysfunctional breathing since this condition is mainly characterized by anatomical and/or physiological alterations.

## Psychosomatic theory

**Figure 1:**
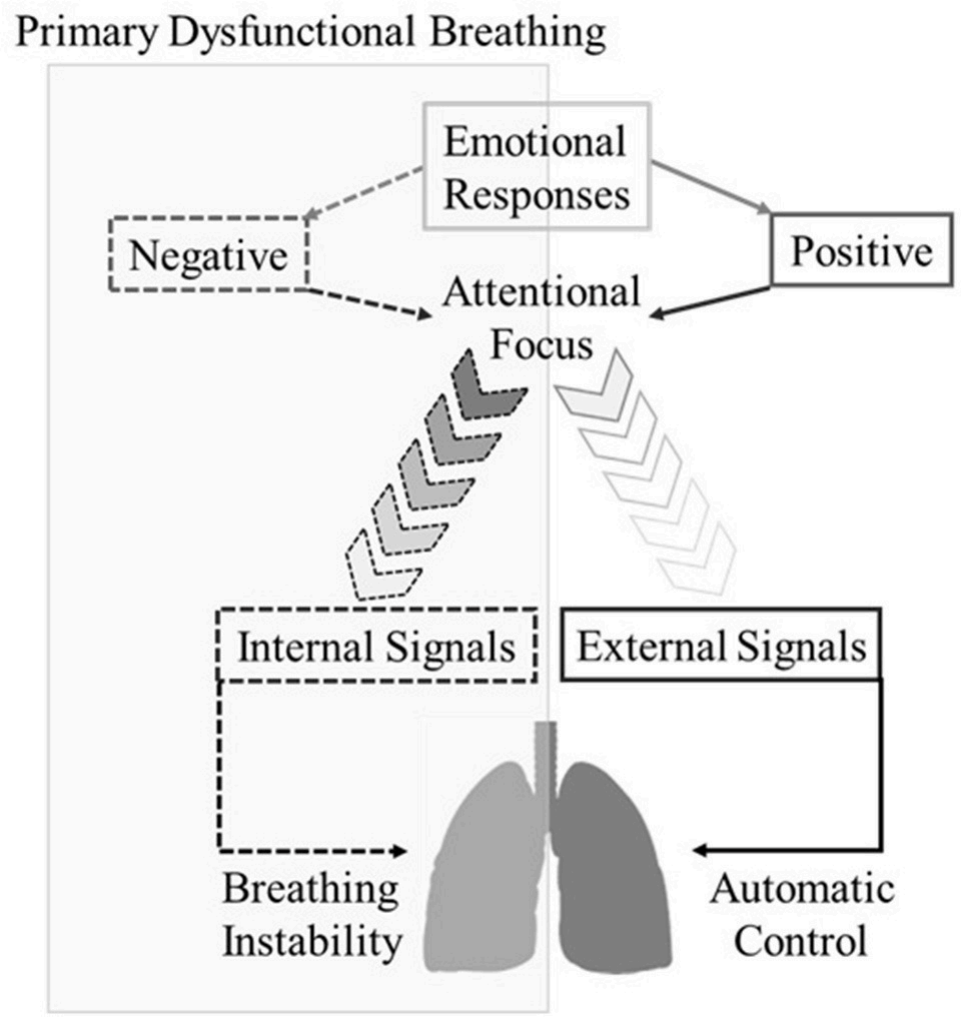
Diagrammatic representation of the psychosomatic theory of primary dysfunctional breathing.

The psychosomatic theory of PDB proposed here (see Figure 1) was developed as a means by which to explain complex psychophysiological phenomena that commonly occur prior to and during irregular breathing episodes. The conceptual framework provided in this article indicates that excessive interoceptive awareness (i.e., thinking too much about bodily sensations) could lead to detrimental effects on breathing stability. This may happen because respiration does not require any form of conscious control. For example, during exercise, the respiratory rate and tidal volume will naturally increase in order to supply the working muscles with sufficient oxygen. However, it is noteworthy that the automatic control of respiration can be overridden by conscious control. In other words, humans are able to up-/down-regulate the respiratory activity consciously. This is due to the fact that skeletal muscles control the movements of the rib cage (i.e., voluntary control of the somatic nervous system). In theory, this biomechanical mechanism could be used to monitor and counteract the negative effects of stress and anxiety, e.g., using breathing exercises (Hagman et al., 2011; van der Zwan et al., 2015). Unfortunately, patients with PDB appear to encounter difficulties while trying to reallocate attentional focus from their breathing to external stimuli. This means that attentional control may be partially compromised in patients with PDB. The theoretical model presented here integrates concepts from different realms of knowledge, such as cognitive psychology, respiratory physiology, and behavioral medicine. Parenthetical examples and/or illustrative description have been used to facilitate the reader's comprehension.

Figure 1 illustrates a sequence of events that may explain erratic breathing episodes in individuals with PDB. The interoceptive sensory system is composed by receptors located throughout the human body (e.g., skeletal muscles and heart). This system emits signals to the brain, which then processes the information and enables us to interpret our physiological status, e.g., limb discomfort (Herbert and Pollatos, 2012). We suggest that the effects of the interoceptive sensory system on the attention and emotion are enhanced in patients with PDB. In such instances, it appears that individuals with PDB tend to redirect their attentional focus inwardly (e.g. movements of the rib cage) whenever stress and anxiety arise (Yamaguchi and Onoda, 2012). This could potentially represent a protective mechanism that individuals with PDB utilize in order to *erroneously* prevent the processing of negative thoughts (cf. Murakami et al., 2015). In such instances, excessive interoceptive awareness can force the central motor command to instinctively control the organism by monitoring the electrical signals emitted to the musculature (Paulus, 2013). However, the correct time and intensity of the contraction that is deemed necessary to supply the organism with oxygen is generally imposed by energy expenditure, and the brain calculates it in an automatic manner (see Pollatos et al., 2007 for a discussion on interoceptive awareness and trait anxiety). Therefore, when individuals attempt to consciously replicate the movements of the rib cage, they usually miscalculate the exact periods of inhalation and exhalation, which could lead to changes in the respiratory cycle (e.g., periods of hyperventilation, deep sigh and/or breath holding). Also, given the precise synchronization of the physiological systems, changes in breathing pattern could potentially lead to subsequent modulations in cardiac and brain activity. In such instances, patients with PDB appear to encounter difficulties in supressing negative emotional responses, which prevents them from re-directing attention back to exteroceptive sensory cues, i.e., overactive monitoring; (cf. Yoris et al., 2017). This psychological mechanism has the potential to initiate domino reactions that subsequently lead to periods of erratic breathing episodes, more negative affective responses and associative thoughts (i.e., a closed loop process). It is also noteworthy that redirecting attention internally is not deemed to be an emotional/psychological deficiency. On the contrary, a number of associative techniques (e.g., breathing meditation) have been extensively used in the fields of exercise and physiotherapy to optimize the neural control of working muscles and prevent external influences from disrupting task performance. However, individuals with PDB might present with a particular form of overactive monitoring that compromises the normal function of the respiratory system.

## Attentional switching

For most people, switching attention back from internal sensory information (e.g., movements of the rib cage) to external influences (e.g., environmental sensory signals) is relatively easy and does not require extreme effort. However, this article has shown how the attentional control and emotional regulation of patients with PDB could be somehow compromised (cf. Lattimore et al., 2017). This psychophysiological mechanism could initiate cascade reactions that would subsequently lead to periods of erratic breathing patterns (see Figure 1). It is important to emphasize that the theoretical model proposed in this article may not be sufficiently sensitive to account for the onset and persistence of symptoms of PDB in all patients. A wide range of emotional triggers can also reawaken long-term memories linked with traumatizing experiences, and subsequently lead to the erratic breathing episodes that are commonly observed in patients with PDB, such as breath-holding, mouth breathing and hyperventilation (Gilbert, 1998; Nardi et al., 2009).

Future research is necessary to test the theoretical propositions of this paper by investigating the attentional and emotional mechanisms that underlie the negative effects of PDB on peripheral physiological responses. Practitioners are also encouraged to apply non-invasive methods of sensory stimulation such as music and videos during erratic breathing episodes to partially assuage the negative bodily sensations that are commonly experienced by patients with PDB, e.g., breathlessness (for details, see Bigliassi, 2015). Audio-visual stimulation has the potential to counteract the acute effects of irregular breathing patterns by distracting patients and increasing the number of external sensory signals that enter focal awareness (Rejeski, 1985). These signals seem to compete for information processing and partially suppress internal association (Hernández-Peón et al., 1961). Meditation techniques (e.g., mindfulness) can also be used as long-term interventions to train individuals how to better control attentional responses and overpower negative emotional reactions (e.g., Bergen-Cico and Cheon, 2014; Kangasniemi et al., 2014).

Finally, breathing retraining programmes (e.g., the Buteyko method) might have the potential to rearrange the autonomous control of respiratory muscles and help patients re-establish a regular breathing pattern. Accordingly, the methods proposed in this paper, such as breathing retraining, mindfulness, music and videos, can be used as valuable tools to test the psychosomatic theory and provide health professionals with effective techniques to counteract the negative effects of PDB. It is time to consider, along with systemic symptoms, the neuropsychological mechanisms that underlie PDB and investigate the topic from a different angle. Brain assessment techniques such as electroencephalography (EEG), functional magnetic resonance imaging (*f* MRI) and functional near-infrared spectroscopy (*f* NIRS) can be used to shine new light on the underlying mechanisms of this respiratory disorder and provide clinicians with more efficient strategies to diagnose and treat PDB.

## Author contributions

The contribution of the authors was as follows: MB and LV conceived the need to write this opinion paper. MB and LV wrote and prepared the manuscript for submission with significant assistance from MJ, AH, and CC. MJ, AH, and CC provided essential input to the idea and writing of the paper. All authors certify to have participated sufficiently in the work to take public responsibility for the appropriateness of the content hereby written.

### Conflict of interest statement

The authors declare that the research was conducted in the absence of any commercial or financial relationships that could be construed as a potential conflict of interest.
